# The Remarkable Mechanism of Prostaglandin E_2_ on Synaptic Plasticity

**Published:** 2008-05-09

**Authors:** Yukio Akaneya

**Affiliations:** Division of Neurophysiology, Department of Neuroscience, Osaka University Graduate School of Medicine, 2-2 Yamadaoka, Suita 565-0871 Japan

**Keywords:** prostaglandin, LTP, visual cortex, cAMP, synaptic plasticity

## Abstract

Prostanoids have a broad spectrum of biological activities in a variety of organs including the brain. However, their effects on synaptic plasticity in the brain, which have been recently revealed, are ambiguous in comparison to those in the other organs. Prostaglandin E_2_ (PGE_2_) is a prostanoid produced from arachidonic acid in the cellular membrane, and knowledge about its functions is increasing. Recently, a novel function of PGE_2_ in the brain has shed light on aspects of synaptic plasticity such as long-term potentiation (LTP). More recently, we have proposed a hypothesis for the mechanisms of this PGE_2_-related form of synaptic plasticity in the visual cortex. This involves the dynamics of two subtypes of PGE_2_ receptors that have opposing functions in intracellular signal transduction. Consequently, mechanisms that increase the level of cyclic AMP in the cytosol may explain for the mechanisms of LTP in the visual cortex. The current notion of bidirectional trafficking of PGE_2_ receptors under this hypothesis is reminiscent of the “silent synapse” mechanism of LTP on the trafficking of the AMPA receptors between the membrane and cytosol. Moreover, we propose the hypothesis that PGE_2_ acts as a “post-to-postsynaptic messenger” for the induction of LTP in the visual cortex. This review describes a complex mode of action of PGE_2_ receptors in synaptic plasticity in the brain.

## Production of PGE_2_

Prostanoids are classified into prostaglandins and the thromboxanes. Prostaglandins consist of prostaglandin (PG)D_2_, PGE_2_, PGF_2a_, and PGI_2_, whereas thromboxanes consist of thromboxane A_2_ (TxA2_2_). All of these products are metabolites of arachidonic acid, which originates from the phospholipid components of the cell membrane. Arachidonic acid is converted to PGG_2_ by the cyclooxygenases, which comprise cyclooxygenase(COX)-1, -2, and 3 (for review, see Schwab et al. 2003). COX-1 is ubiquitous and COX-3 is a splice variant of COX-1, whereas COX-2 is inducible in the brain by the activity in the physiological or pathological condition. For example, the level of COX-2 mRNA is enhanced in long-term potentiation (LTP), which is a form of synaptic plasticity and is defined as the persistent enhancement of synaptic transmission efficacy (Yamagata et al. 1993; Chen and Bazan, 2005). Because PGG2_2_ is remarkably unstable, PGG_2_ is immediately converted to PGH_2_. Then PGH_2_ is metabolized to a specific prostanoid by a corresponding synthase; for example, PGE_2_ is produced by the PGE synthase.

In the prostanoids, PGG_2_, PGH_2_, PGI_2_ and TxA_2_ are degraded into inactive products within several minutes even under physiological conditions, whereas in *in vivo* the other prostaglandins are immediately inactivated through circulation in the lungs, where oxidative stress is strong. It is believed that because of the chemical and biological instability of prostanoids, the area, where the activity of prostanoids is essential for performing and maintaining the physiological conditions is believed to be restricted around the region where they prostanoids are produced. Prostanoids are hydrophobic, because they are synthesized from fatty acids in the cell membrane. This property allows them to pass through the membrane and reach neighboring cells. Thus, prostanoids are able to exert their functions approximately in spite of their short lifetime. This is consistent with the fact that the prostanoids which are produced in the brain do not circulate in the blood, and yet can act in the neighboring subcellular regions, such as synapses.

## Receptors for PGE_2_

Four subtypes of receptor for PGE_2_ have been identified: EP1, EP2, EP3, and EP4 (EP1–4) (for review, see Narumiya et al. 1999; Narumiya and FitzGeraldl, 2001). These four receptor subtypes are expressed in different levels in the brain as well as in the other organs.

Although EP1 mRNA is distributed mainly in the kidneys, lungs, and stomach, EP1 is also found in the neurons in thalamus and dorsal root ganglia (DRG) (Watabe et al. 1993). However, a recent study showed that the levels of EP1 mRNA are the highest in the parietal cortex and cerebellum and that the protein level of EP1 is high in cerebellum (Candelario-Jalil et al. 2005). Although EP2 is the least abundant among the PGE_2_ receptor subtypes in the brain, its expression can be induced by stimulation with lipopolysaccharide (LPS) and gonadotropin (Katsuyama et al. 1998). A study involving Northern blot analysis and *in situ* hybridizatuion showed that EP3 is expressed abundantly and widely in the brain (Sugimoto et al. 1992). EP3 mRNA is expressed in the neurons of the cerebral cortex, hippocampus, thalamus, midbrain, and lower brain stem. However, EP4 mRNA is expressed only in the neurons of the DRG (Sugimoto et al. 1994; Oida et al. 1995; Burks et al. 2007).

Binding of agonists to these receptor subtypes induces signal transduction via secondary messengers, such as cyclic AMP (cAMP), in a G protein-coupled manner. The activation of EP1 elevates the levels of intracellular inositol phosphate and Ca^2+^ (Watabe et al. 1993), whereas the activation of EP2 or EP4 stimulates adenylate cyclase, resulting in increased levels of intracellular cAMP (Jumblatt and Paterson, 1991). Conversely, activation of EP3 inhibits adenylate cyclase (Sugimoto et al. 1992). Recently, we have shown that synaptic plasticity in the visual cortex is deeply involved in these opposing effects of EP2 and EP3 on the production of cAMP. This is a good way to acquire the sufficient cAMP for the induction of LTP (see below).

## Functions of PGE_2_ in the Brain

PGE_2_ is a mediator of fever induction (Milton and Wendlandt 1970). It acts as a final mediator in the induction of fever by the pyrogens acting on the organum vasculosum of the lamina terminalis (OVLT) of the third lateral ventricle (Saper and Breder, 1994). An *in situ* hybridization study showed that EP3 mRNA is expressed at a particularly high level in the regions surrounding the OVLT (Sugimoto et al. 1994). Circulating pyrogens that may generate PGE_2_ have an easy access to the OVLT because it lacks blood-brain barrier. There have been increasing numbers of knockout (KO) studies on the four receptor subtypes, EP1–4, and in one of these studies EP3-deficient mice showed no febrile responses to any of the pyrogens tested (Ushikubi et al. 1998).

PGE_2_ and PGI_2_ promote bradykinin-induced pain (Katori et al. 1986). However, a KO study on the receptors involved revealed that this type of pain may require receptors for PGI_2_ rather than PGE_2_ (Murata et al. 1997). It is also known that PGE_2_ acts on the spinal dorsal horn to produce pain (Malmberg et al. 1995; Pitcher and Henry, 1999). Outside the central and peripheral nervous systems, it is known that PGE_2_ promotes the coagulation of platelets via EP3 (Ma et al. 2001), bone resorption and formation (Sakuma et al. 2000; Miyata et al. 2000; Li et al. 2000), cancer and carcinogenesis (Watanabe et al. 1999), hypertension (Kennedy et al. 1999; Tilley et al. 1999) and cardiovascular functions (Audoly et al. 1999; Zhang et al. 2000). Thus, PGE_2_ exerts a variety of functions in the body, including the brain. However, it is only recently that PGE_2_ has been shown to have functions in synaptic plasticity.

## Knockdown for EP1–4

It is important to evaluate the functions of known and novel genes in order to understand the mechanisms involved. Genetic manipulation by KO is a useful tool to characterize a target gene. Indeed, KO studies in mice for EP1–4 in mice KO studies have been reported recently. The most eminent feature of genetic manipulation by KO is its specificity for the target gene, i.e. the genes whose expression is to be deduced. This is the most important reason why the method has become so popular and has produced so much information over the last decade. However, gene KO has shortcomings that hinder its use in ordinary laboratories. These include (1) the unpredictable effects of the knocked-out protein on other proteins that might in turn influence some other proteins especially during development. This makes it difficult to know whether the results obtained are caused because of the absence of the target protein or other proteins; (2) the high frequency of death before birth or during early postnatal development, resulting in reduced opportunity to conduct experiments; (3) the limitation of the knocked-out region (in most cases, the whole body). The effect of ordinary KO manipulation is systemic and begins at fertilization in almost all cases. It is difficult to predict the location and timing of the effect of the KO desired; (4) the fact that in most cases KO experiments are limited to mice, resulting in the restriction on the use of antibodies, DNA probes and so on; and (5) the large expenditure, labor, and time required to establish the KO model. With respect to reason (1), it is difficult to know whether the results obtained are caused by the absence of the target protein or by the absence or lack of other proteins. With respect to the reason (3), the effect of ordinary KO manipulation is systemic and begins at fertilization in almost all cases. It is difficult to predict exactly the location and timing for the effects of the KO desired. Nonetheless, KO manipulations have been used to analyze the function of the target proteins by many researchers. This is mainly because of the specificity of targeting, which presumably avoids none-specific actions, such as pharmacological actions.

An alternative way to inhibit the target expression is by RNA interference (RNAi), in which stretches of 21–23 nucleotides of RNA, termed short (or small) interfering RNA (siRNA), are used to inhibit the expression of the target mRNA, resulting in the inhibition of the target protein (Fire et al. 1998). The mechanisms of RNAi is believed to be as follows. The enzyme Dicer, a member of the RNase III family of nucleases, specifically cleaves double-stranded RNAs to form siRNAs. The siRNAs subsequently bind to RNAi-induced silencing protein complex (RISC), which contains an endo-nuclease; this is followed by targeting the perfectly complementary mRNA and cleaving the target within the sequences covered by the siRNA. Guidance of RISC to the target mRNA is highly sequence-specific; a difference of only one or two nucleotides in the targeting recognition sequence interferes with the RNAi process. The primary siRNA-template RNA can be used to support *de novo* RNA synthesis by an RNA-dependent RNA polymerase, resulting in the amplification of RNA. With respect to target specificity and the efficacy of maintenance of RNAi, the method using siRNA has great advantages over the use of the conventional antisense oligonucleotides to knock down the target.

For introducing exogenous molecules such as proteins, RNA, and DNA into the cells, electroporation is a convenient and efficient method (for review, see Neumann et al. 1999). The mechanisms comprises two steps: first, electrical shocks applied at the membrane produce pores that last for tens of seconds; external molecules that are negatively charged then enter the cytosol by electrophoresis. The efficiency of expression of exogenous genes has been reported to be 10–100% according to the parameters of electric impulses used. In most cases, however, the targets of the *in vivo* electroporation are whole organs, such as the liver and brain in the embryonic stages of the chick and mouse. Electroporation at the embryonic stage offers the possibility of influencing the destiny of the proteins other than the target as mentioned above.

To resolve the defects of systemic KO or knockdown manipulation mentioned above, we developed a novel method, termed RNAi-induced silencing by local electroporation, RISLE (Akaneya et al. 2005). It involves electroporation with the two needle electrodes after injecting siRNA into the target area of the brain to introduce the siRNA. In a recent paper, we showed that the target gene expression at the site where siRNA is injected between the two electrodes is exclusively knocked down, and that the effect is target-specific. We also found that, when using a combination of siRNA with local electroporation, the expression of certain proteins is significantly reduced in the restricted regions, such as the visual cortex and the CA1 region of the hippocampus, and use of the technique does not induce pathological changes. Moreover, we have shown that this model is available for *in vitro* and *in vivo* experiments such as electrophysiology.

Using the RISLE method, we knocked down EP1–4 in rats and analyzed the mechanisms of synaptic plasticity in the visual cortex with these knockdown rats (Akaneya and Tsumoto, 2006). We found that both EP2 and EP3, but not EP1 or EP4, are involved in the LTP in the visual cortex.

## Differences in Site of Action of PGE_2_ between Hippocampus and Visual Cortex

Previous papers have reported the effect of PGE_2_ on synaptic plasticity. However, there is controversy regarding the sites of action of PGE_2_, i.e. presynaptic or postsynaptic sites of neurons. First, it has been reported that PGE_2_ increases excitatory postsynaptic potentials (EPSPs) in hippocampal slices and frequency, and that this action is via the EP2 which is located at the presynaptic sites (Sang et al. 2005; Zhu et al. 2005). This suggests the presynaptic action of PGE_2_. Immediately after this publication, we showed that PGE_2_ increases the magnitude of theta-burst stimulation (TBS; 5 stimulus trains at the interval of 10s, each train consisting of 10 bursts at 5 Hz and each burst consisting of 4 pulses at 100 Hz) induced LTP in the slices of rat visual cortex. Glutamate released from presynaptic sites by TBS activates *N*-methyl-D-aspartate receptor (NMDAR) at postsynaptic sites, followed by a Ca^2+^ influx. This activates Ca^2+^-dependent cPLA_2_, which then produces arachidonic acid from the membrane lipid substrate, in addition to the activation of Ca^2+^/calmudulin-dependent protein kinase II (CaMKII). Arachidonic acid is metabolized to PGH_2_ by COX-2 that has been activated by TBS concomitantly (Akaneya and Tsumoto, 2006). This action is mediated via EP2 at the postsynaptic sites in neurons of the visual cortex, which is inconsistent with the previous study in the hippocampus. The two studies depend mainly on the electrophysiological approach and the immunocytochemical analysis of the pre- and postsynaptic marker proteins, synaptophysin and postsynaptic density protein-95 (PSD-95), respectively. PGE_2_ increased the synaptic stimulus-evoked amplitudes of EPSPs in hippocampal slices and the frequency of miniature excitatory postsynaptic currents (mEPSCs) in hippocampal neurons in culture (Sang et al. 2005). In contrast, PGE_2_ has no significant effects on the paired-pulse ratio in the visual cortex slices or on the release of glutamate from synaptoneurosomes of the visual cortex (Akaneya and Tsumoto, 2006). Immunocytochemical analysis of the hippocampal neurons in culture revealed that EP2 highly colocalized with synaptophysin (71.0%), but partially with PSD-95 (14.2%), whereas EP3 partially colocalized with PSD-95 (45.3%) or synaptophysin (38.7%) (Zhu et al. 2005). The former and the other results suggest the pre- and postsynaptic actions of PGE_2_, respectively. In cultures of neurons from the visual cortex, however, both EP2 and EP3 are highly colocalized with PSD-95 (EP2, 71%; EP3, 82%), but less colocalized with synaptophysin (EP2, 25%; EP3, 13%) (Akaneya and Tsumoto, 2006). The difference may be due to the subcellular location of EP2, i.e. at the presynaptic site in the hippocampus and at the postsynaptic site in visual cortex. Moreover, both studies showed that EP2 and EP3, but not EP1 or EP4, are abundantly expressed in the hippocampus or visual cortex. This suggests the implication of EP2 and EP3 in the physiological functions of these regions of the brain. Another common point between these two studies is that PGE_2_ is synthesized at the postsynaptic sites by COX-2. Other papers have reported various findings about neuronal and synaptic functions which are common to the hippocampus and visual cortex. Further investigation, such as electron microscopic studies of the subcellular distribution of EP receptor subtypes, especially for EP2, is necessary in order to resolve this inconsistency.

## A Hypothesis: Bidirectional Trafficking of PGE_2_ Receptor Subtypes

**Figure 1:**
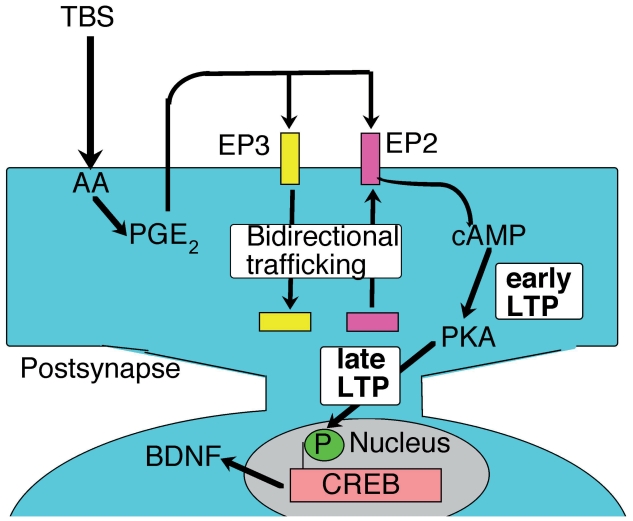
Hypothetical mechanism of PGE_2_-mediated LTP in the visual cortex. TBS produces arachidonic acid (AA) from the membrane lipid substrate. AA is metabolized to PGH_2_ by COX-2 that has been activated concomitantly by TBS. PGH_2_ is converted immediately to PGE_2_ by PGE_2_ synthase. EP2 translocates from the cytosol to the membrane, simultaneously, with the translocation of EP3 from the membrane to the cytosol. The PGE_2_ that is generated spreads from postsynaptic sites into the synaptic cleft, where it activates EP2 at the postsynaptic membrane, resulting in the production of cAMP. Subsequently, cAMP activates PKA, which in turn activates CREB in the nucleus of postsynaptic cells in the visual cortex. This activation of CREB may induce the synthesis of proteins such as BDNF, which is involved in the L-LTP.

LTP comprises early- and late-LTP (E-LTP and L-LTP, respectively) (Frey et al. 1993; Abel et al. 1997; Huang et al. 2004). E-LTP lasts for a few hours after its initiation, and L-LTP lasts for hours, days or even months after the end of E-LTP. As mentioned above, we have shown that PGE_2_ increases the magnitude of both E- and L-LTP in the visual cortex (Akaneya and Tsumoto, 2006). This involves interesting and efficient trafficking of EP2 and EP3, both of which are located at the postsynaptic sites in the neurons of the visual cortex. We propose the following to account for the mechanism of PGE_2_-related LTP. In the stationary state, EP2 and EP3 are mainly located in the cytosol and at the membrane, respectively (Fig. 1). The TBS that induces LTP causes bidirectional trafficking of EP2 and EP3 into the membrane and the cytosol, respectively, which results in an increase in EP2 and a decrease in EP3 at the membrane. Simultaneously, TBS produces PGE_2_ by COX-2. PGE_2_-stimulation of EP2 or EP3 induces an increase or a decrease in the level of cAMP, respectively, in the cytosol via adenyl cyclase (Jumblatt and Paterson, 1991; Sugimoto et al. 1992). This shift in the composition of the PGE_2_ receptor subtypes at the membrane, i.e. the predominant location of EP2, can easily induce the production of a large amount of cAMP in the cytosol. Consequently, cAMP activates cAMP-dependent protein kinase (PKA), which may in turn activate cAMP response element binding protein (CREB), a transcription factor, in the nucleus. The maintenance of L-LTP depends on protein synthesis (Frey et al. 1993; Abel et al. 1997; Huang et al. 2004). This new protein synthesis resulting from the activation of CREB may therefore be involved in L-LTP. It is known that synaptogenesis occurs during L-LTP, and these newly synthesized proteins may be used for the formation of synapses.

A pharmacological analysis revealed that CaMKII may be involved in the trafficking of EP2 from the cytosol to the membrane, but the mechanism of EP3-trafficking is unknown (Akaneya and Tsumoto, 2006). The dynamic force causing this trafficking might be provided by as yet unknown EP2- and EP3-interacting proteins, similar to the AMPA receptor-interacting proteins involved in the so-called silent synapse mechanisms (Harris and Lim, 2001; Barry and Ziff, 2002; Collingridge et al. 2004). This bidirectional trafficking of EP2 and EP3 is similar to the trafficking of AMPA receptors in LTP.

A candidate for the product formed as a consequence of the activation of CREB is brain-derived neurotrophic factor (BDNF). Our previous work has shown that BDNF increases the efficacy of synaptic transmission and TBS-induced LTP in the visual cortex (Akaneya et al. 1997). Moreover, quantitative PCR analysis has shown that TBS induces the generation of BDNF mRNA in the visual cortex (Akaneya and Tsumoto, 2006). Taking these results together, we surmise that PGE_2_ has a direct action on E-LTP and an indirect action on L-LTP via the production of BDNF.

## A Hypothesis: A Candidate for “Post-to-Postsynaptic Messenger”

The induction of LTP requires the activation of NMDA receptor at the postsynaptic cells. The maintenance of LTP requires, at least in part, a change in presynaptic function. In other words, some known or unknown factors, the so-called retrograde messengers, must be transmitted from the postsynaptic to the presynaptic cells, where they must affect presynaptic functions. Many candidates for these retrograde messengers have been proposed (for review, see Williams, 1996). It is possible to say that in the hippocampus PGE_2_ works as a retrograde messenger, because PGE_2_ is produced at the postsynaptic site and then diffuses across the postsynaptic membrane to the presynaptic site where EP2 is distributed (Chen et al. 2005; Sang et al. 2005; Zhu et al. 2005). In the visual cortex, on the other hand, it is possible to say that, as a new conception, PGE_2_ works as a “post-to-postsynaptic messenger,” because PGE_2_ is produced at the postsynaptic site and then passes across the membrane into the synaptic cleft where PGE_2_ activates EP2 at the postsynaptic membrane (Akaneya and Tsumoto, 2006).

## Conclusion

PGE_2_ is generated from membrane phospholipids, which is ubiquitous in the central nervous systems. The prostanoids that is produced from this source is not only PGE_2_. Therefore, it is important to regulate the production of PGE_2_ for the maintenance and successful accomplishment of function. Here I want to propose “an intricate machinery” of neurons to yield the energy that is necessary for ordinary synaptic functions. Bidirectional trafficking of the two subtypes, EP2 and EP3, which have reciprocal actions is one of the ways by which synaptic functions may be regulated. In future, further elucidation of the mechanisms of action of PGE_2_ in the synaptic plasticity, in combination with new methods for introducing agents into the restricted brain regions, such as RISLE, may come to be useful for developing new therapies for psychoneurological diseases such as dementia and cerebrovascular diseases.
